# Integrated multi-omics analyses reveal the altered transcriptomic characteristics of pulmonary macrophages in immunocompromised hosts with *Pneumocystis pneumonia*

**DOI:** 10.3389/fimmu.2023.1179094

**Published:** 2023-06-09

**Authors:** Yawen Wang, Kang Li, Weichao Zhao, Yalan Liu, Ting Li, Hu-Qin Yang, Zhaohui Tong, Nan Song

**Affiliations:** ^1^ Department of Respiratory and Critical Care Medicine, Beijing Institute of Respiratory Medicine and Beijing Chao-Yang Hospital, Capital Medical University, Beijing, China; ^2^ Department of Respiratory Medicine, Strategic Support Force Medical Center, Beijing, China; ^3^ Medical Research Center, Beijing Institute of Respiratory Medicine and Beijing Chao-Yang Hospital, Capital Medical University, Beijing, China

**Keywords:** *Pneumocystis pneumonia*, glucocorticoids, single-cell RNA sequencing, macrophages, immunosuppression

## Abstract

**Introduction:**

With the extensive use of immunosuppressants, immunosuppression-associated pneumonitis including *Pneumocystis jirovecii pneumonia* (PCP) has received increasing attention. Though aberrant adaptive immunity has been considered as a key reason for opportunistic infections, the characteristics of innate immunity in these immunocompromised hosts remain unclear.

**Methods:**

In this study, wild type C57BL/6 mice or dexamethasone-treated mice were injected with or without *Pneumocystis*. Bronchoalveolar lavage fluids (BALFs) were harvested for the multiplex cytokine and metabolomics analysis. The single-cell RNA sequencing (scRNA-seq) of indicated lung tissues or BALFs was performed to decipher the macrophages heterogeneity. Mice lung tissues were further analyzed via quantitative polymerase chain reaction (qPCR) or immunohistochemical staining.

**Results:**

We found that the secretion of both pro-inflammatory cytokines and metabolites in the *Pneumocystis*-infected mice are impaired by glucocorticoids. By scRNA-seq, we identified seven subpopulations of macrophages in mice lung tissues. Among them, a group of Mmp12^+^ macrophages is enriched in the immunocompetent mice with *Pneumocystis* infection. Pseudotime trajectory showed that these Mmp12^+^ macrophages are differentiated from Ly6c^+^ classical monocytes, and highly express pro-inflammatory cytokines elevated in BALFs of *Pneumocystis*-infected mice. *In vitro*, we confirmed that dexamethasone impairs the expression of *Lif*, *Il1b*, *Il6* and *Tnf*, as well as the fungal killing capacity of alveolar macrophage (AM)-like cells. Moreover, in patients with PCP, we found a group of macrophages resembled the aforementioned Mmp12^+^ macrophages, and these macrophages are inhibited in the patient receiving glucocorticoid treatment. Additionally, dexamethasone simultaneously impaired the functional integrity of resident AMs and downregulated the level of lysophosphatidylcholine, leading to the suppressed antifungal capacities.

**Conclusion:**

We reported a group of Mmp12^+^ macrophages conferring protection during *Pneumocystis* infection, which can be dampened by glucocorticoids. This study provides multiple resources for understanding the heterogeneity and metabolic changes of innate immunity in immunocompromised hosts, and also suggests that the loss of Mmp12^+^ macrophages population contributes to the pathogenesis of immunosuppression-associated pneumonitis.

## 1 Introduction

*Pneumocystis*, an opportunistic fungal organism, causes *Pneumocystis jirovecii pneumonia* (PCP) in immunocompromised individuals, including those HIV or non-HIV infected patients (1). Recently, the prevalence of PCP gradually increases due to the increasing use of immunosuppressive therapies, such as the glucocorticoids treatment (2, 3).

Adaptive immune responses play a critical role in the protection against *Pneumocystis* infection in immunocompetent hosts (4). We previously demonstrated that B cells play a critical role in the regulation of T-helper (Th) cells during *Pneumocystis* infection (5), and suppression of B cell immunity by glucocorticoid treatment can lead to PCP development (6). Programmed death 1 (PD-1) deficiency can also promote the phagocytic capacity of macrophages and Th1/Th17 response, and improve *Pneumocystis* clearance (7). Furthermore, we have also elucidated the signatures of T cells and B cells repertoire profiling and their dynamics during *Pneumocystis* infection (8, 9), confirming the contribution of adaptive immunity in controlling PCP.

Dexamethasone (DEX) is one of the most frequently used glucocorticoids in clinical research studies (10, 11). DEX treatment has been proved beneficial in patients with inflammatory diseases (12). Mechanistically, DEX can exert anti-inflammatory function through either genomic or non-genomic actions, either by regulating gene expression or altering the cation transport, respectively (12, 13). DEX exerts potent regulatory effects on both innate and adaptive immunity. However, how glucocorticoids regulate immune response upon *Pneumocystis* infection, as well as the underlying mechanism, remains unclear. Enhancing the innate immunity against the pathogenic microbes without influencing the therapeutic purpose is a clinically desirable status.

In this study, we employed single-cell RNA sequencing (scRNA-seq) and metabolomics to map the characteristics of heterogeneous pulmonary macrophages in both immunocompetent and immunocompromised hosts. We revealed significantly different transcriptomics patterns between the two immune states. From intensive investigation, we defined a population of recruited Mmp12^+^ macrophages featured by actively promoting host defense against pathogens challenge. We also showed that the resident alveolar macrophages (AMs) are composed of both pro- and anti-inflammatory subpopulations. Moreover, our data suggested that the downregulation of granulocyte/macrophage colony-stimulating factor (GM-CSF) may contribute to the pathogenesis of PCP in immunocompromised hosts, which provides insights into designing therapeutic strategies for immunocompromised patients by targeting macrophages.

## 2 Results

### 2.1 Glucocorticoid treatment impairs the secretion of pro-inflammatory cytokines in the bronchoalveolar lavage fluids of *Pneumocystis*-infected mice

**Figure 1:**
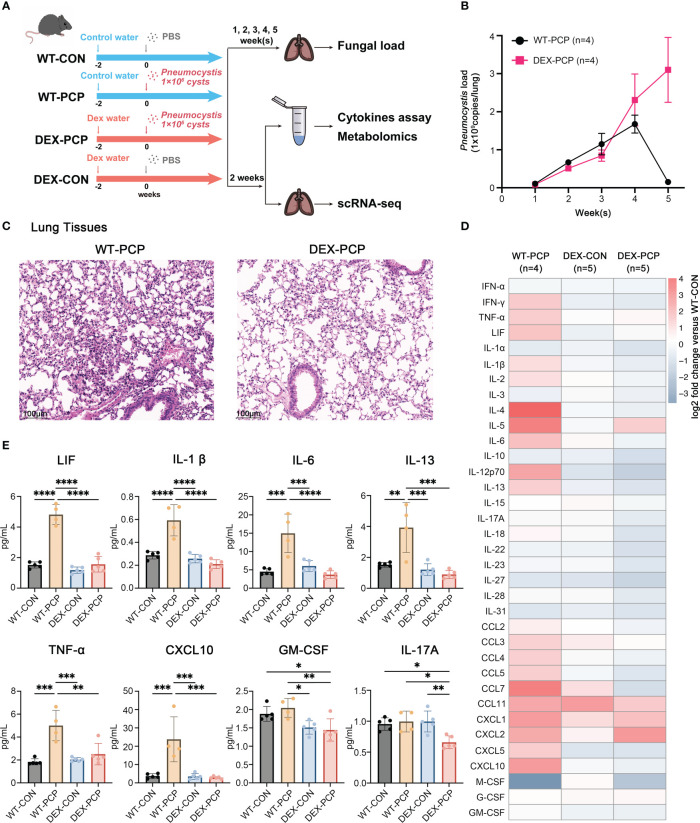
Dexamethasone impairs *Pneumocystis* clearance and cytokines secretion in mice. **(A)** Schematic diagram for the infection schedule and overall study design. Mice were exposed to DEX or not for 2 weeks and then were intratracheally instilled with *Pneumocystis* of 1×10^6^ cysts. These mice were sacrificed at different time points and lung tissues or BALFs were then collected for analysis of *Pneumocystis* burden, cytokines, metabolites or scRNA-seq. **(B)** Mice were incubated with *Pneumocystis* intratracheally and monitored for *Pneumocystis* burden in WT-PCP and DEX-PCP mice over the 5-week course of infection (n = 4 per group). **(C)** The pathological characteristics demonstrated by H&E staining in WT-PCP and DEX-PCP mice at 2 weeks post infection. **(D)** Heatmap for multiplex analysis of cytokines in BALFs at 2 weeks post infection for indicated groups of mice (n = 4 or 5 per group). Values represent log_2_ fold change *versus* the WT-CON group. **(E)** Bar plots showing selected differentially expressed cytokines in BALFs from **(D)**. In **(B)**, the results were presented as means ± SE of 4 mice per group in each experiment, performed in triplicate. In **(E)**, the results were presented as means ± SD of 4 or 5 mice per group. Comparisons were evaluated by one-way ANOVA for multiple comparisons. *p < 0.05, **p < 0.01, ***p < 0.001, ****p < 0.0001.

We first assessed the altered immune responses in immunocompromised hosts during *Pneumocystis pneumonia*. To do so, a pharmacologically immunosuppressed mouse model was established by DEX feeding for 2 weeks (**Figure 1A**). Both DEX-treated and immunocompetent mice were then intratracheally injected with *Pneumocystis murina* (*P. murina*) at the dose of 1×10^6^ cysts. Control mice were administered with phosphate buffer solution (PBS). The *Pneumocystis* burden of infected immunocompetent mice (WT-PCP) and DEX-induced immunocompromised mice (DEX-PCP) were investigated at indicated time points. As shown in **Figure 1B**, the fungal load peak was observed at 4 weeks post infection in the immunocompetent mice, and a significant reduction of the fungal load was found after 5 weeks, consistent with our previous report (9). In contrast, the fungal load gradually increased in the DEX-treated group post infection, suggesting that the immune responses required for controlling *Pneumocystis* growth were impaired in these immunocompromised mice.

We further performed hematoxylin and eosin (H&E) staining to analyze the pathological changes in the lungs of both groups at 2 weeks post infection. Strikingly, although the fungal burden were comparable between DEX-treated and non-treated mice, the amount of recruited immune cells were more evident in immunocompetent mice, compared with that in the immunocompromised mice. These data indicated that DEX treatment significantly interfered with the early immune response against *Pneumocystis* infection (**Figure 1C**).

As cytokines and chemokines are critical for recruiting immune cells to the sites of infection, we next determined the secretion of these factors in both immune states following infection. To do so, bronchoalveolar lavage fluids (BALFs) from indicated groups were collected and analyzed by cytokine multiplex analysis (**Figures 1D, E**). We found that many canonical pro-inflammatory cytokines including leukemia inhibitory factor (LIF), TNF-α, IL-1β, IL-6, CXCL10, *etc.*, were highly elevated in WT-PCP mice relative to uninfected control, while DEX treatment significantly impaired the secretion of these factors. Besides, the level of GM-CSF, critical for the differentiation of the myeloid lineage, was also decreased in DEX-PCP mice *versus* WT-PCP counterparts.

In addition, as shown in a previous study (14), *Pneumocystis* infection can induce both Th2 and Th17 responses, stimulate the secretion of IL-13 and IL-17A, and further facilitate the formation of inducible bronchus associated lymphoid tissue (iBALT) in a *Cxcl13*-dependent manner. Accordingly, we further compared the levels of IL-13 and IL-17A and found the secretion were both decreased in the BALFs of DEX-PCP group, compared with indicated control group (**Figure 1E**), which suggests the impaired iBALT formation in the immunocomprised mice. Taken together, our results demonstrate that immunocompetent mice can control and eliminate *Pneumocystis via* inducing a series of immune responses, while glucocorticoid treatment leads to an ineffective fungal clearance in *Pneumocystis*-infected mice, probably due to the defects in eliciting protective immunity, stimulating iBALT formation and secreting pro-inflammatory factors.

### 2.2 Pro-inflammatory metabolites are decreased in the immunosuppressed hosts with *Pneumocystis* infection

**Figure 2:**
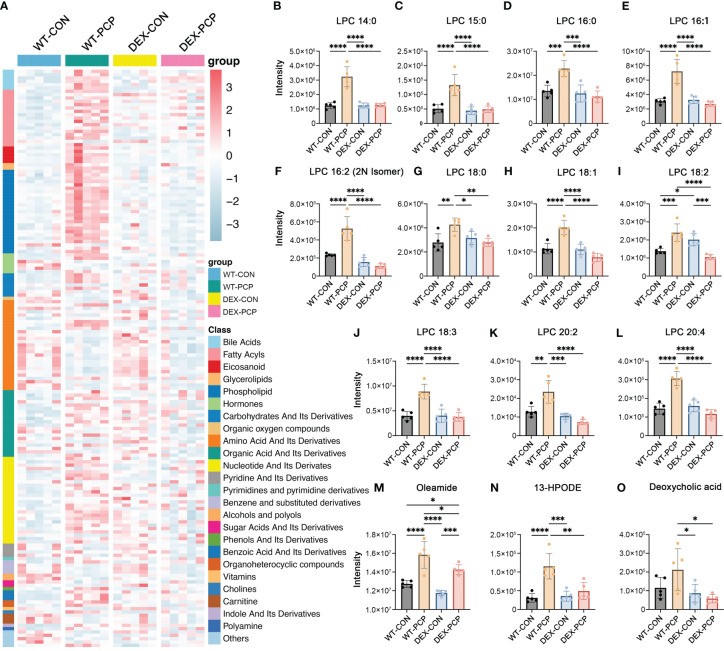
Metabolomic features of BALFs derived from mice with *Pneumocystis* infection. **(A)** Heatmap showing the differentially expressed metabolites for WT-PCP *versus* WT-CON and DEX-PCP. The result showed significant changes in the levels of bile acids, fatty acyls, eicosanoid, glycerolipids and phospholipids. **(B–L)** Bar plots showing differentially expressed metabolites LPC belonging to phospholipids from **(A)**. **(M–O)** Bar plots showing differentially expressed metabolites oleamide (fatty acyls), 13-HPODE (eicosanoid) and deoxycholic acid (bile acid) from **(A)**. The results were presented as means ± SD of 5 mice per group. Comparisons were evaluated by one-way ANOVA for multiple comparisons. *p < 0.05, **p < 0.01, ***p < 0.001, ****p < 0.0001.

Recent studies indicate that cellular metabolism influences the response of host immunity (15). To gain an insight into the metabolic changes caused by DEX treatment and *Pneumocystis* infection, BALFs from 4 groups of mice (5 mice per group) were collected for quantitative metabolomics analysis. After data preprocessing and annotation, 611 metabolites were included in the dataset. Here we mainly focused on the metabolites that were significantly altered due to both *Pneumocystis* infection and DEX treatment (**Figure 2A**, **Supplementary Data 1**). Our data showed that a series of metabolites including phospholipids, bile acids, fatty acyls, eicosanoid, *etc.* were dramatically increased in the mice of WT-PCP group, compared with the control group (WT-CON), while significantly blocked by DEX treatment. As shown, lysophosphatidylcholines (LPCs), a class of phospholipids derived from phosphatidylcholine, was prominent accumulated in the WT-PCP mice than DEX-PCP group (**Figures 2B–L**). Considering that LPCs have been recognized as a class of metabolites positively associated with inflammation, and can promote macrophage polarization (16), the upregulation of LPCs may function as critical metabolites that modulate host immunity against infection. Besides, the changes of several pro-inflammatory metabolites including oleamide (fatty acyl), 13-HPODE (eicosanoid) and deoxycholic acid (bile acid) were also observed during infection and DEX treatment (**Figures 2M–O**). Together with previous observations that DEX treatment leads to a decreased cytokine production and restricted antimicrobial responses, these data suggest that DEX treatment significantly alters the host immunity, and prompt us to perform an in-depth exploration of the DEX-induced changes of the host immune microenvironment.

### 2.3 Single cell analysis reveals an aberrant immune cell composition in immunosuppressive *Pneumocystis*-infected mice

**Figure 3:**
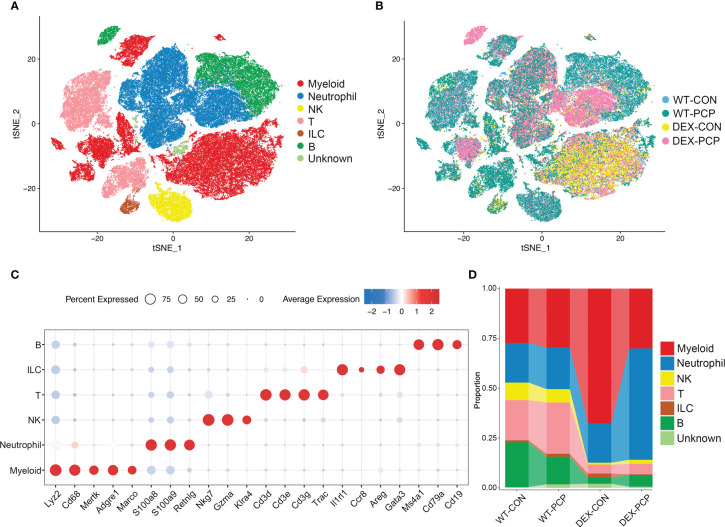
Single-cell analysis reveals aberrant immune cell composition in immunosuppressive *Pneumocystis*-infected mice. **(A)** t-SNE plot for 84149 immune cells, color-coded by cell types. **(B)** t-SNE plot for 84149 immune cells, color-coded by groups with 21861 cells from WT-CON group, 22822 cells from WT-PCP group, 14728 cells form DEX-CON group and 24738 cells from DEX-PCP group (n =3 per group). **(C)** Dot plot showing the average expression of canonical markers of each cell type. Data were colored based on the gene expression levels. **(D)** Stacked bar plot showing the average proportion of each immune cell type from each group.

To decipher how DEX exerted influence on the altered secretion of cytokines and metabolites during infection, we performed scRNA-seq on cell suspensions derived from the lung tissues of indicated groups of mice (3 mice per group) at 2 weeks post *Pneumocystis* infection, respectively (**Figure 1A**). After quality filtering, we obtained 122785 cells with 400-6000 genes detected per cell, including 84149 immune cells and 38636 non-immune cells, respectively. Here, we mainly focused on the immune cell population, and performed subsequent analysis involving principal component analysis, dimensionality reduction and clustering. Clustering analysis of these immune cells identified 24 distinct clusters consisting of myeloid cells (*Cd68*, *Lyz2*), neutrophils (*S100a8*, *S100a9*, *Retnlg*), natural killer (NK) cells (*Nkg7*, *Klra4*), T cells (*Cd3d*, *Cd3e*, *Cd3g*), innate lymphoid cells (ILC) (*Il1rl1*, *Areg*) and B cells (*Ms4a1*, *Cd79a*), identified by canonical signature genes (**Figure 3A**). Among these cells, myeloid cells showed a specific enrichment pattern in different groups (**Figures 3B–D**). In DEX-treated group, the total amount of lymphoid cells including NK, T, ILC and B cells were significantly decreased, compared with immunocompetent mice. Immune cells in the lungs of DEX-treated mice were mainly composed of myeloid cells and neutrophils, and data showed that myeloid cells are significantly increased in DEX-treated group (**Figure 3D**). Therefore, we mainly focused on the anti-fungal role of myeloid cells in our present study.

**Figure 4:**
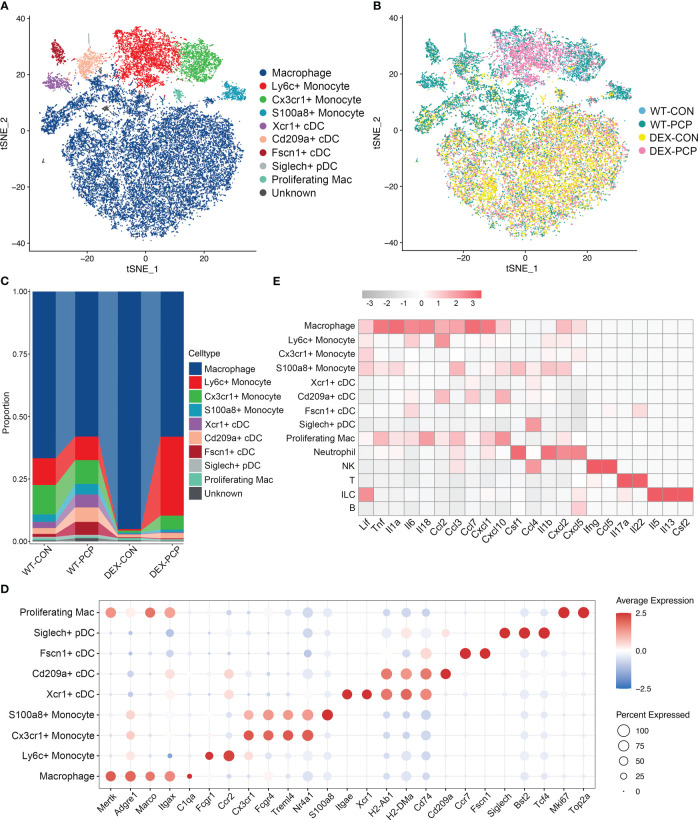
Dissection of myeloid cells showing anti-microbial functions of macrophages during *Pneumocystis* infection. **(A)** t-SNE plot for 30150 myeloid cells, color-coded by cell types. **(B)** t-SNE plot for 30150 myeloid cells, color-coded by groups with 5963 cells from WT-CON group, 6776 cells from WT-PCP group, 9981 cells form DEX-CON group and 7430 cells from DEX-PCP group. **(C)** Stacked bar plot showing the average proportion of each myeloid cell type from each group, with macrophages accounting for a large proportion. **(D)** Dot plot of the average expression of canonical markers of each cell type. Data were colored based on the gene expression levels. **(E)** Heatmap of gene expression of GM-CSF and pro-inflammatory cytokines detected in BALFs in **Figure 1** for each subtype from all samples.

Then, we re-clustered the total myeloid cells to further explore the dynamically transcriptional alterations induced by *Pneumocystis* infection after DEX treatment. By assigning to known myeloid cell types, these cells were designated as 9 distinct subpopulations representing monocytes, dendritic cells (DCs) and macrophages, according to identified marker genes (**Figure 4A**). Of note, the macrophage population was more abundant in immunosuppressive group (**Figures 4B–D**), and highly expressed the mRNA of most of cytokines elevated in BALFs derived from WT-PCP mice (**Figure 4E**), suggesting that macrophages play important roles in the protection against *Pneumocystis* infection in the immunosuppressed hosts. In addition, we also showed that the percent of several DCs subtypes were upregulated in the WT-PCP group, while evidently decreased in the DEX-PCP group. As one of the critical antigen presenting cell types in the lungs, DCs can interact with *Pneumocystis*, and induce humoral and Th2 cellular immune responses (17). Thus, our results suggested these DCs may also confer protection during *Pneumocystis* infection in immunocompetent mice. However, in the DEX-treated mice, lymphocytes are significantly suppressed, therefore macrophages may play more essential roles.

### 2.4 Recruited monocyte-derived Mmp12^+^ macrophages confer protection during *Pneumocystis* infection

**Figure 5:**
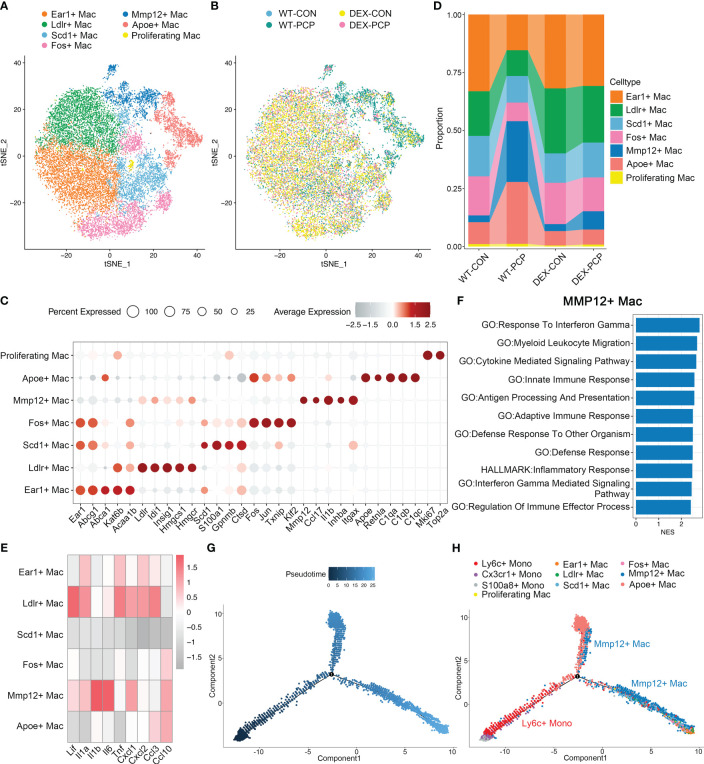
Mmp12^+^ macrophages are enriched during *Pneumocystis* infection. **(A)** t-SNE plot for 21720 macrophages, color-coded by cell types. **(B)** t-SNE plot of 21720 macrophages, color-coded by groups with 3979 cells from WT-CON group, 3934 cells from WT-PCP group, 9490 cells form DEX-CON group and 4317 cells from DEX-PCP group. **(C)** Dot plot of the average expression of highly expressed genes for each subtype. Data were colored based on the expression levels. **(D)** Stacked bar plot showing the average proportion of each macrophage cell type from each group, with Mmp12^+^ macrophages varying obviously. **(E)** Heatmap of genes expression of pro-inflammatory cytokines detected in BALFs in **Figure 1** for each macrophage subtype further suggesting the protective function of Mmp12^+^ macrophages. **(F)** GSEA analysis using DEGs of Mmp12^+^ macrophages *versus* other macrophages to explore the functions of Mmp12^+^ macrophages. **(G, H)**. Differentiation trajectory inferred of Mmp12^+^ macrophage *via* Monocle2 using all monocytes and macrophages from WT-PCP group, colored by pseudotime in **(G)** and cell type in **(H)**.

Macrophages are the main component of myeloid cells, and have been considered to represent a heterogeneous population (18). However, this heterogeneity has not been elucidated in *Pneumocystis pneumonia*. Given this notion, we next probed the diversity and complexity of macrophages in the *Pneumocystis*-infected mice. The 21720 macrophages derived from the lung tissues of aforementioned mice were clustered into seven subsets, consisting of Ear1^+^ macrophages, Ldlr^+^ macrophages, Scd1^+^ macrophages, Fos^+^ macrophages, Mmp12^+^ macrophages, Apoe^+^ macrophages and proliferating macrophages, according to the differentially expressed genes (DEGs) (**Figures 5A–C**, **Supplementary Data 2**). Among them, the Apoe^+^ macrophages, highly expressing *Apoe*, *C1qa*, *C1qb* and *C1qc*, represent an identical population recently termed as interstitial macrophages (IMs) (19, 20).

The presence of immunosuppressant DEX and *Pneumocystis* dramatically altered the composition of macrophages in lungs (**Figure 5D**). Especially, the proportion of Mmp12^+^ macrophages was significantly increased after *Pneumocystis* infection, while dramatically decreased upon DEX treatment. Further, these Mmp12^+^ macrophages highly expressed pro-inflammatory genes including *Lif*, *Il1b*, *Il6* and *Cxcl10* (**Figure 5E**). To gain a comprehensive insight into the functional characteristics of Mmp12^+^ macrophages, we identified DEGs and performed gene set enrichment analysis (GSEA) against pathways in the gene ontology (GO) as well as Kyoto Encyclopedia of Genes and Genomes (KEGG) database. The results showed that the DEGs of Mmp12^+^ macrophages were enriched in inflammatory response, defense response and cytokine mediated signaling pathway, indicating their critical role in conferring protection against infections (**Figure 5F**).

Alveolar macrophages are the major population of the tissue resident macrophages, mainly derived from embryonic precursors and capable of self-renewal (21, 22). These cells can also be replenished by monocyte-derived macrophages following an infection or the sterile inflammation. To explore the cell fate transition of these macrophage populations, we utilized Monocle2 to infer cell trajectories (**Figures 5G, H**). Pseudotime analysis showed that four subsets including Ear1^+^ macrophages, Ldlr^+^ macrophages, Scd1^+^ macrophages, and Fos^+^ macrophages, represent resident AMs derived from embryonic precursors. Mmp12^+^ macrophages, highly expressing *Itgax*, originated from Ly6c^+^ classical monocytes. After recruitment, these Mmp12^+^ cells may develop into resident macrophages, suggesting that these Mmp12^+^ cells play a pro-inflammatory role in protection against *Pneumocystis* infection.

### 2.5 Dexamethasone impairs the differentiation of Mmp12^+^ macrophages

**Figure 6:**
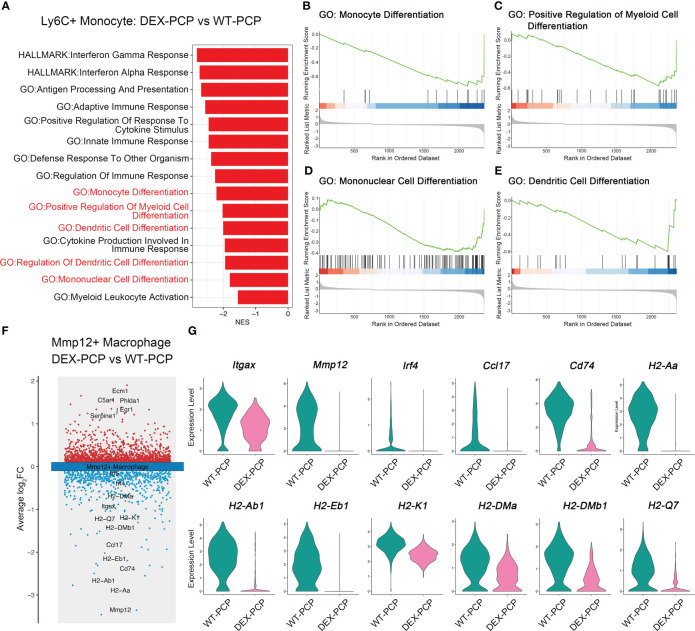
Dexamethasone impairs the differentiation of Mmp12^+^ macrophages from Ly6c^+^ monocytes. **(A)** GSEA analysis of DEGs of Ly6c^+^ monocytes from DEX-PCP *versus* WT-PCP group. **(B–E)** Selected enriched pathways in **(A)** were displayed. **(F)** Volcano plot showing the DEGs of Mmp12^+^ macrophages from DEX-PCP *versus* WT-PCP group, revealing genes responsive to GM-CSF were significantly downregulated in DEX-PCP mice. **(G)** Violin plots showing the expression level for selected genes esponsive to GM-CSF in Mmp12^+^ macrophages from DEX-PCP *versus* WT-PCP.

In inflammatory diseases, myeloid cells were the key population potentially responsive to GM-CSF (23). Our data showed that the decreased expression of GM-CSF was observed in the DEX-PCP mice, which inhibited the monocyte to macrophage differentiation. Combining the aforementioned observations of a relatively higher proportion of Ly6c^+^ monocytes and a lower proportion of Mmp12^+^ macrophages in DEX-PCP mice, we wondered whether DEX hampers the differentiation and development of Mmp12^+^ macrophages. The gene expression profiles of Ly6c^+^ monocytes were compared between DEX-PCP and WT-PCP groups. GSEA revealed that the DEX significantly downregulates multiple pathways including monocyte differentiation, mononuclear cell differentiation, *etc.* (**Figures 6A–E**)

The comparison of Mmp12^+^ macrophages in DEX-PCP group *versus* WT-PCP group was also performed. We found that the expression of *Itgax*, *Mmp12*, *Irf4*, *Ccl17*, *Cd74* and genes encoding MHC class II were all markedly reduced in the Mmp12^+^ macrophages of DEX-PCP mice (**Figures 6F, G**). In agreement with this observation, previous reports suggested that interferon regulatory factor 4 (IRF4), a key downstream of GM-CSF, can upregulate the expression of CCL17 and genes encoding MHC class II (23, 24). It has also been revealed that GM-CSF facilitates the CD11c expression, which is required for the differentiation of monocytes into alveolar macrophages (25, 26). Consistently, as shown in our data, *Csf2* (encoding GM-CSF) is mainly expressed by ILCs in WT-PCP group (**Figure 4E**), while in DEX-treated mice, the ILC population was significantly inhibited, suggesting the reason for the lower expression level of this cytokine in immunocompromised hosts.

**Figure 7:**
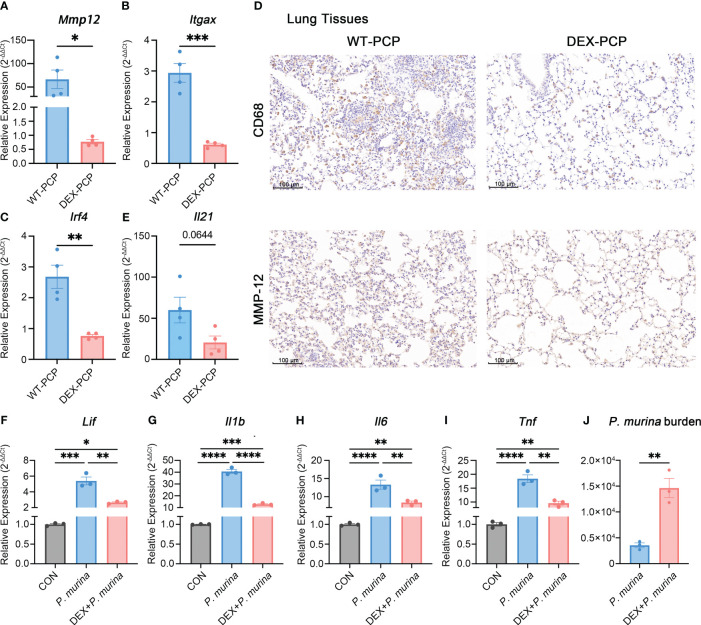
Dexamethasone treatment reduces the number of Mmp12^+^ macrophages and impairs the fungal killing capacity of macrophages. **(A–C)** Analysis of *Mmp12*, *Itgax* and *Irf4* mRNA levels in WT-PCP and DEX-PCP mice lungs by qPCR. **(D)** Detection of CD68 and MMP-12 in lung tissue sections by immunohistochemistry. Representative images were shown. **(E)** Analysis of *Il21* mRNA level in WT-PCP and DEX-PCP mice lungs by qPCR. In **(A–C, E)** data are presented as the means ± SE fold change in *Mmp12*, *Itgax*, *Irf4* and *Il21* mRNA levels normalized to the β-actin mRNA compared with WT-CON mice. Comparisons were evaluated by unpaired Student’s *t* test. **(F–I)** Analysis of *Lif*, *Il1b*, *Il6* and *Tnf* mRNA levels in AM-like cells at 2 hr post *P. murina* incubation following DEX-treatment. Data are presented as means ± SE and multiple comparisons were evaluated by one-way ANOVA. **(J)** Detection of *P. murina* burden at 24 hr post *P. murina* incubation following DEX-treatment. Data are presented as means ± SE and comparisons were evaluated by unpaired Student’s *t* test. *p < 0.05, **p < 0.01, ***p < 0.001, ****p < 0.0001.

To further confirm this notion, we assessed the expression of *Mmp12*, *Itgax* and *Irf4* in DEX-treated or untreated mice at 2 weeks post *Pneumocystis* infection. The quantitative polymerase chain reaction (qPCR) studies on lung tissues showed that the mRNA expression of *Mmp12*, *Itgax* and *Irf4* were markedly decreased in DEX-PCP group (**Figures 7A–C**). Moreover, the immunohistochemical examination showed negligible staining of MMP-12 and macrophages maker CD68 in DEX-PCP mice lung tissues, compared with the WT-PCP group (**Figure 7D**). These data further confirmed that DEX treatment inhibits the differentiation of Mmp12^+^ macrophage during *Pneumocystis* infection. Additionally, the obvious downregulation of genes encoding MHC class II in Mmp12^+^ macrophages of DEX-PCP mice may subsequently contribute to the dysfunction of CD4^+^ T cells. It has been reported that IL-21 signaling is necessary for CD4^+^ T cell effector responses. Thus, we further investigated the *Il21* expression and found that it may be downregulated in DEX-PCP mice, compared with WT-PCP counterpart (**Figure 7E**). These results confirm that DEX treatment decreases the expression of GM-CSF, then reduces the capacity of differentiation and maturity of myeloid cells and influences T cell immunity *via* multiple pathways including the downregulation of *Il21* signaling.

Moreover, we investigated the role of DEX in the fungal killing capacity of macrophages *in vitro*. Firstly, AM-like cells were generated from mouse bone marrow with the treatment of GM-CSF, TGF-β, and peroxisome proliferator–activated receptor γ (PPAR-γ) agonist as described (27). These AM-like cells were then treated with or without DEX before *P. murina* infection. The expression of pro-inflammatory cytokines and the degree of *P. murina* burden were monitored by qPCR. We found that DEX treatment impairs the *P. murina*-induced expression of *Lif*, *Il1b*, *Il6* and *Tnf* (**Figures 7F–I**), and leads to a significant increase in fungal burden in these AM-like cells (**Figure 7J**). Together with our previous *in vivo* results (**Figure 1B**), these data further confirmed that DEX can dampen the anti-fungal immune responses.

### 2.6 Group 2 macrophages in patients with PCP resembles Mmp12^+^ macrophages in mice

To validate our findings in human samples, we performed scRNA-seq for the BALFs derived from two patients diagnosed with *Pneumocystis jirovecii pneumonia*. Both patients are immunocompromised, while receiving different immunosuppressants, Methotrexate/Tripterygium Glycosides (Non-glucocorticoids, designated as Patient #1) and Prednisone (Glucocorticoids, designated as Patient #2), respectively (**Supplementary Data 3**). The scRNA-seq data of BALF cells from three healthy donors were downloaded from the GEO database (GSE145926). After quality filtering, 38203 cells from five samples were obtained for further analysis. Principal component analysis, dimensionality reduction and clustering were conducted as described in **Figure 3**. These cells were clustered into myeloid cells/neutrophils, T cells, B cells, epithelial cells and proliferating cells according to canonical signature genes (**Supplementary Figure 1**).

**Figure 8:**
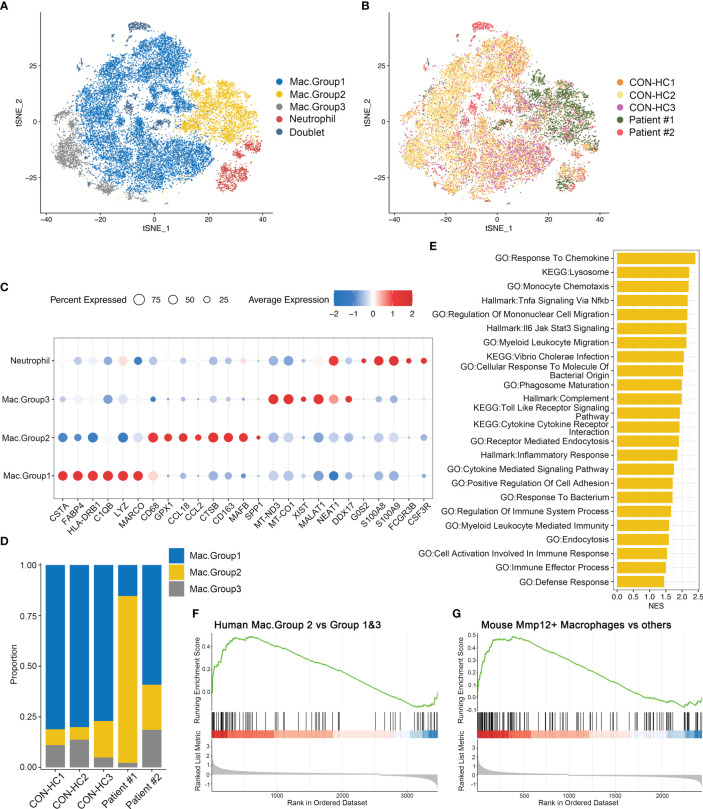
Group 2 macrophages in patients with PCP resemble Mmp12^+^ macrophages in mice. **(A)** t-SNE plot for 27118 myeloid cells and neutrophils, color-coded by cell types. **(B)** t-SNE plot for 27118 cells, color-coded by donors with 8888 cells from CON-HC1, 8880 cells from CON-HC2, 4032 cells from CON-HC3, 3853 cells from Patient #1 ((Non-glucocorticoids) and 1465 cells from Patient #2 (Glucocorticoids). **(C)** Dot plot of the average expression of highly expressed genes for each subtype. Data were colored based on the expression levels. **(D)** Stacked bar plot showing the average proportion of each macrophage group from each donor, with group 2 macrophage varying obviously. **(E)** GSEA analysis of DEGs of group 2 macrophages *versus* group 1&3 macrophages to explore the functions of group 2 macrophages. **(F)** GSEA analysis of DEGs of group 2 macrophages *versus* group 1&3 macrophages using signatures of Mmp12^+^ macrophages in mouse dataset. **(G)** GSEA analysis of DEGs of mouse Mmp12^+^ macrophages *versus* other macrophages using signatures of group 2 macrophages in human dataset.

We next re-clustered total myeloid cells to dissect their heterogeneity. Based on the gene expression patterns, we designated 3 groups of macrophages (**Figures 8A–C**). Of note, group 2 macrophages (Mac.Group2) characterized by highly MAFB expression, which is essential for human monocytes-derived macrophages differentiation (28), suggesting that these cells originate from monocytes (29). Moreover, this macrophage population is specifically enriched in the BALFs of patient #1, but not the patient receiving glucocorticoids treatment (**Figure 8D**). We then investigated DEGs of group 2 macrophages *versus* group 1&3 and performed GSEA analysis. Consistent with the data obtained in Mmp12^+^ macrophage (**Figure 5F**), both the pro-inflammatory and anti-microbial pathways, including response to chemokine, inflammatory response, defense response, *etc.* are also upregulated in group 2 macrophages (**Figure 8E**). By comparing the transcriptomic features of group 2 macrophages with Mmp12^+^ macrophages in mice, we found that Mac.Group2 exhibited the characteristic signatures similar to Mmp12^+^ macrophages, and vice versa (**Figures 8F, G**), indicating the functional similarity of the two groups of macrophages. These data further support the existence of a group of anti-infectious macrophages during *Pneumocystis* infection, which may be inhibited by glucocorticoids treatment.

### 2.7 Dexamethasone upregulates the expression of *Lpcat3* in resident AMs and lowers the level of lysophosphatidylcholine in BALFs

**Figure 9:**
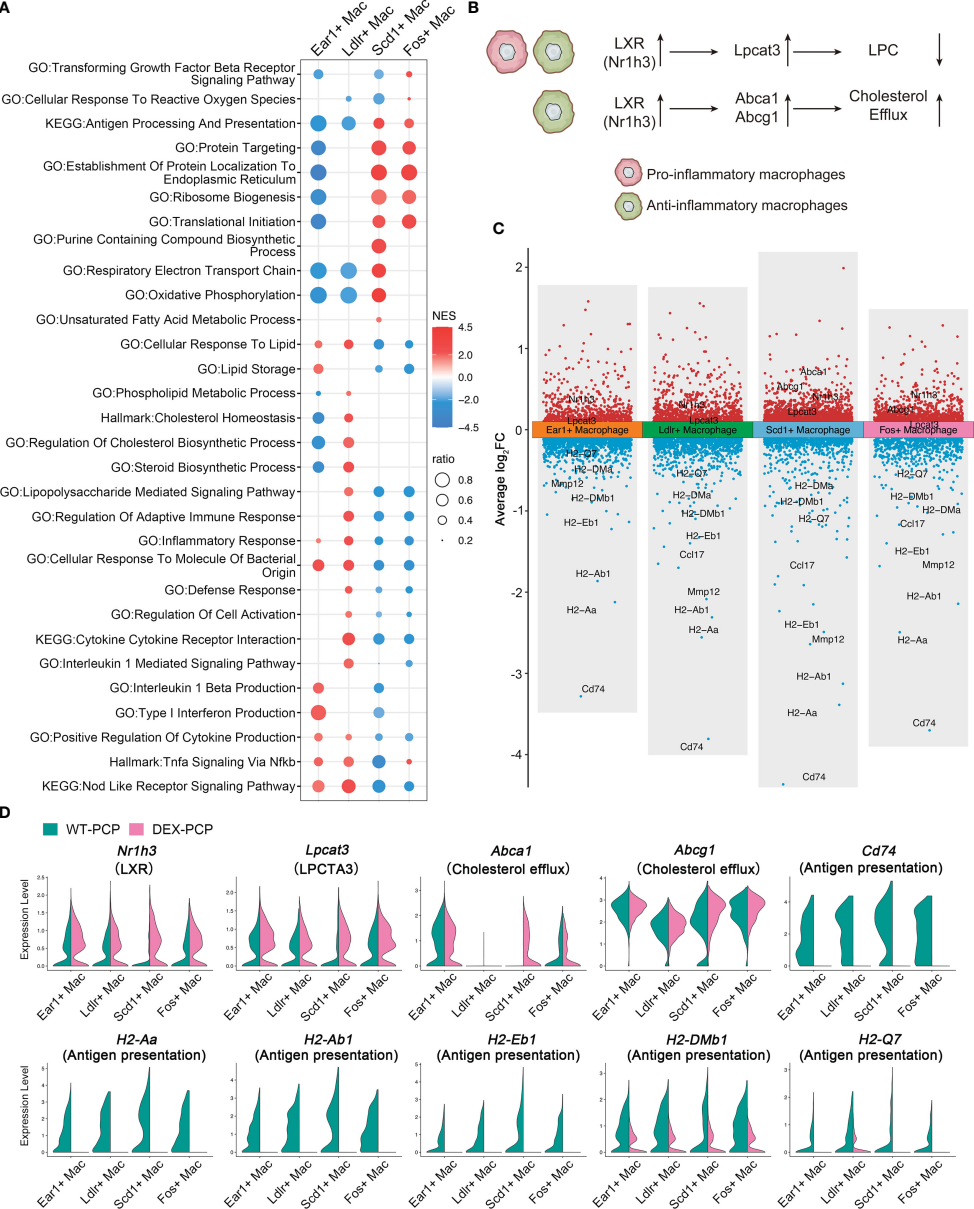
Dexamethasone treatment leads to the dysfunction of four resident AMs subtypes. **(A)** Dot plot for enriched pathways *via* GSEA analysis of each resident AMs subtype *versus* other macrophages, showing two distinct gene expression patterns. **(B)** Schematic plot showing impacts DEX extered on different resident AMs subtypes. **(C)** Volcano plot showing the DEGs for each resident AMs subtype in DEX-PCP *versus* WT-PCP counterpart. **(D)** Violin plots showing expression levels of selected genes involved in LPCs, cholesterol efflux and responsive to GM-CSF in each resident AMs subtype from DEX-PCP and WT-PCP group.

We next focused on 4 other subsets of resident AMs in mice, and compared the expression levels of their functional pathways (**Figure 9A**). We found that these subsets exhibited distinct gene expression patterns. The Ear1^+^ macrophages and Ldlr^+^ macrophages are enriched in ‘positive regulation of cytokine production’, ‘cellular response to molecule of bacterial origin’, ‘inflammatory response’, *etc.*, indicating that they may exert an anti-infectious activity. In contrast, the other two subsets, Scd1^+^ macrophages and Fos^+^ macrophages, showed a completely opposite gene expression pattern. The expression of cytokines and chemokines genes involved in inflammation were further explored among all identified macrophage groups. We found that Ear1^+^ macrophages and Ldlr^+^ macrophages are characterized by the expression of pro-inflammatory factors such as *Lif*, *Tnf*, *Il1a*, *Il6*, *Cxcl1* and *Cxcl2*, suggesting their pro-inflammatory phenotype (**Figure 5E**).

**Figure 10:**
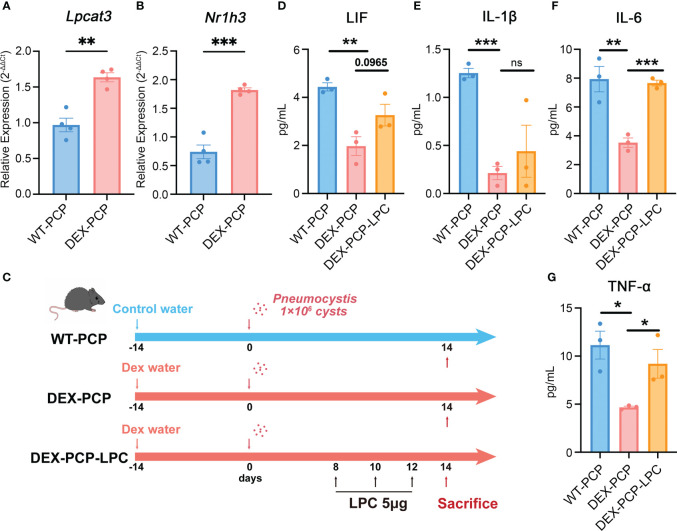
LPC treatment partially rescues the pro-inflammatory cytokines expression in dexamethasone-treated mice. **(A, B)** Analysis of *Lpcat3* and *Nr1h3* mRNA levels in WT-PCP and DEX-PCP mice lungs by qPCR. **(C)** Schematic plot for the LPC exposure schedule and study design. DEX-treated immunosuppressive mice were challenged with *Pneumocystis* of 1×10^6^ cysts. Then these mice were intratracheally administered LPC 5 μg each time on day 8, day 10 and day 12 post infection. Mice were sacrificed on day 14 post infection and BALFs were collected to analyze the levels of cytokines. **(D–G)** Bar plots showing the levels of LIF, IL-1β, IL-6 and TNF-α in BALFs from indicated group of mice in **(C)** (n = 3 per group). In **(A, B)**, data are presented as the means ± SE fold change in *Lpcat3* and *Nr1h3* mRNA level normalized to the β-actin mRNA compared with WT-CON mice. In **(D–G)**, data are presented as the means ± SE. Comparisons were evaluated by unpaired Student’s *t* test. *p < 0.05, **p < 0.01, ***p < 0.001, ****p < 0.0001.

As mentioned in **Figure 2**, LPCs were a major class of pro-inflammatory metabolites exhibiting a dramatic alteration between WT-PCP and DEX-PCP group. LPCs could be converted into phosphatidylcholine by lysophosphatidylcholine acyltransferases (LPCATs) (16). Among them, LPCAT3, downstream of Liver X receptors (LXRs), is specially expressed in murine macrophages (30, 31). Accordingly, we determined these LXR-related gene expression in the 4 subsets of AMs before and after DEX treatment, and found that *Nr1h3* (encoding LXRs) and *Lpcat3* (encoding LPCAT3) were both elevated in the samples of DEX-PCP group, compared with WT-PCP counterpart, which may lead to the decreased level of LPCs (**Figures 9B–D**). We next validated the expression of *Lpcat3* and *Nr1h3* in the DEX-treated or untreated mice lungs at 2 weeks post *Pneumocystis* infection using qPCR (**Figures 10A, B**). The data showed that the expression levels of both *Lpcat3* and *Nr1h3* are markedly elevated in the DEX-PCP group comparing to WT-PCP mice. To further investigate the function of LPC during infection, DEX-treated mice were intranasally administered LPC at indicated time points post *Pneumocystis* challenge (**Figure 10C**). The BALFs were collected and analyzed using cytokine multiplex analysis (**Figures 10D–G**). The data showed that LPC can partially rescue the secretion of pro-inflammatory cytokines, including LIF, IL-1β, IL-6 and TNF-α, in BALFs of DEX-PCP mice.

Furthermore, our data showed that in Scd1^+^ macrophages and Fos^+^ macrophages, the DEX treatment upregulates two LXRs downstream genes *Abca1* and *Abcg1* (**Figures 9B–D**), both of which can promote the efflux of cholesterol to suppress inflammation. Together with the finding that the GM-CSF-induced expression of IRF4 could be inhibited by LXR agonists (32), our data indicate that DEX treatment can trigger a profound immunosuppressive effect in the Scd1^+^ and Fos^+^ resident AMs, which further disrupted the anti-infection immunity of the hosts.

## 3 Discussion

Here, we have conducted a detailed investigation to decipher the influences of glucocorticoids on the heterogeneity of lung macrophages in the context of *Pneumocystis* infection. By using a combination of multiplex cytokine analysis, metabolomics and scRNA-seq, we revealed that DEX impairs the differentiation of the recruited protective monocyte towards Mmp12^+^ macrophages, and dampens the pro-inflammatory function of resident AMs, probably due to the reduction of GM-CSF.

GM-CSF, as a hematopoietic growth factor for myelopoiesis, has also been considered as a cytokine critical for the differentiation of the myeloid lineage (33). GM-CSF also played important roles in regulating host defense against *Pneumocystis* (34). In CD4-deficient mice, the level of GM-CSF in lung tissues is increased after *Pneumocystis* infection. GM-CSF-deficient mice exhibits increased fungal burden and aberrant lung function post infection, due to the decreased phagocytosis and TNF-α production in AMs (34). Glucocorticoids has also been reported to reduce the level of GM-CSF produced in both bronchial epithelial cells (35), airway smooth muscle (36, 37), and monocyte-derived macrophages (38). Moverover, in patients with giant cell arteritis, a systemic granulomatous vasculitis, the GM-CSF level in artery biopsy specimens is decreased after receiving glucocorticoids therapy (39). Together with these reports, we found that the level of GM-CSF in BALFs is much lower in the DEX-treated group than that in control mice, suggesting GM-CSF, which functions as a key driver for activating the pro-inflammatory role of macrophages, may be targeted by glucocorticoids in hosts with *Pneumocystis* infection.

In a study on immunosuppressed organ transplant hosts, Xu et al. showed that GM-CSF selectively restored multiple DEX-suppressed genes, and protected DEX-treated mice from *Salmonella* infection (40). A series of studies have also showed that GM-CSF can reverse the immunosuppressive effects of DEX on bronchoalveolar macrophages and peritoneal macrophages, and rescue the clearance of *Aspergillus conidia* and the production of pro-inflammatory cytokines, involving the activation of NF-κB translocation (41–43). These investigations suggested a potential use of GM-CSF in patients receiving DEX treatment at risk for opportunistic infections. Based on these findings, we further elaborate the detailed effects of glucocorticoids on various macrophages subtypes, and suggest the potential therapeutic use of GM-CSF in improving the resistance of DEX-treated hosts against *Pneumocystis* infections.

Macrophages are the primary phagocytes of the innate immune system with a high degree of plasticity, and understanding the functions of distinct macrophage populations is critical to decipher disease pathogenesis (44). Macrophages in the lung can be broadly divided into AMs, present in the airways or alveoli, and IMs, present in the tissue interstitium (21). These lung macrophages, especially AMs, can function as immune sentinels, and play a critical role in the recognition, phagocytosis, destruction and clearance of lung pathogens such as *Pneumocystis* (45). Moreover, with the deepening study of the polarization of macrophages, researchers have gradually reached a consensus that macrophages are very heterogeneous, and a binary M1/M2 classification is inadequate to capture the complexity (44, 46). For instance, Melgert and her colleagues reported the existence of differentially polarized macrophages during the allergic inflammation of murine lung (47). Currently, several studies have explored the role of macrophage in *Pneumocystis*-infected hosts, while still mainly focused on M1/M2 polarization (48, 49). Better understanding of the heterogeneity of macrophages during *Pneumocystis* infection is still lacking. In the current study, we have explored this issue on the single-cell level, and defined a population of monocyte-derived macrophages with high Mmp12 expression in *Pneumocystis*-infected hosts, which conferred considerable antimicrobial protection. We also validated these data in BALF cells from patients with *Pneumocystis jirovecii pneumonia*. The group 2 macrophages are obviously enriched in pathways related to immune response, and confirmed to have similar transcriptomic profiles to Mmp12^+^ macrophages. In the presence of DEX, this group of recruited macrophages was significantly reduced, associated with the decreased GM-CSF level, suggesting the reason why infection tends to be exacerbated in the glucocorticoids-treated patients.

MMP-12 (also known as macrophage elastase) was expressed in AMs, and first identified as an enzyme possessing elastolytic activity (50, 51). As a pro-inflammatory mediator, aberrant activation of MMP-12 led to multiple diseases progression, including chronic obstructive pulmonary disease (COPD) (52), pneumonia (53), *etc.* Degradation of extracellular matrix components by MMP-12 is critical for the migration of macrophage, as Mmp12^-/-^ macrophages were incapable of penetrating reconstituted basement membranes (54). In a study of colitis, MMP-12, served as a key pathogenic factor, can degrade the basement membrane laminin, therefore facilitating macrophage transmigration across the intestinal tight junctions (55). It has also been shown that GM-CSF treatment can increase the MMP-12 expression in both macrophages and monocyte-derived cells (56, 57). As recently shown in COPD, csGRP78^hi^ AMs, mostly expressed MMP-12, expanded in cigarette smoke (CS)-induced COPD mice and showed the potential to be therapeutic target (52). In atherosclerosis, a subset of Lgals3^-^ macrophages exhibiting highly expressed Mmp12 accumulated in the advanced plaques with pro-inflammatory characteristics (56). Though Nelson et al. have showed that Mmp12-deficient mice were not more susceptible to *Pneumocystis* (58), we considered that the group of macrophages characterized by Mmp12 may function through many other immune mediators other than degrading extracellular matrix.

The immune changes during *Pneumocystis* infection have always been widely concerned. CD4^+^ T cells have been believed to play an essential role in clearing *Pneumocystis*, as demonstrated by HIV-induced immunosuppressive patients with decreased CD4^+^ T cells susceptible to *Pneumocystis* infection (59). Kolls JK and colleagues found that *Pneumocystis* challenge is capable of priming type 2 immunity including Th2 response (60). Simultaneously, anti-infective Th17 response (type 3 immunity) is also induced during *Pneumocystis pneumonia* (61). ILC2 secreting effector cytokines IL-5, IL-13 and ILC3 secreting IL-17, IL-22 participated synergistically in type 2 or 3 immunity respectively (62). In our data, we found that the change of IL-13 in indicated groups were more obvious than IL-17A, and ILC mainly expressed *Il5* and *Il13* in WT-PCP mice. Therefore, type 2 and 3 immunity were both stimulated in WT-PCP mice while ILC2 may play a more important role in the protection against pneumonia. However, our scRNA-seq data showed that not only the lymphocytes, but also the myeloid cells were affected by DEX treatment, suggesting that DEX-induced immunocompromised model may exhibit a severe defect in innate immune responses against *Pneumocystis* infection. Consistently, it has been reported that the prognosis for HIV-negative *Pneumocystis pneumonia* patients is worse than those HIV-infected PCP patients (63).

In conclusion, we first proposed the Mmp12^+^ macrophages featured by conferring considerable protection in the lungs of *Pneumocystis*-infected mice. Treatment of glucocorticoids exerted undesirable effects especially on inhibiting both the development of recruited monocytes and the functional integrity of resident AMs. These findings are expected to become potential targets for treating the infection in immunocompromised individuals.

## 4 Materials and methods

### 4.1 Patients

This study was conducted in conformity to the approved guidelines of the Institutional Review Boards of Beijing Chao-Yang Hospital, Capital Medical University. Two patients definitely diagnosed with PCP (male, mean age, 69 years) were enrolled in present study. Both participants have signed informed consent. BALFs samples derived from two subjects were collected in sterile tubes using standard techniques. The specimens were immediately stored on ice, first filtered through a 0.1 μM cell strainer and then centrifuged at 700 g for 5 min to obtain cell pellets. The resulting pellet was lysed with RBC lysing buffer (BD) to remove red blood cells followed by centrifugation as well as cell counts and viability determination.

The scRNA-seq data of BALF cells from three healthy donors was downloaded from the GEO database (GSE145926).

### 4.2 Mice

Healthy C57BL/6 female mice were purchased from Vital River Laboratory Animal Technology Co., Ltd. (Beijing China) and housed in specific pathogen-free conditions. Mice were randomly grouped into four groups as described previously. DEX-induced immunocompromised mice were given drinking water dissolving 4 mg/L DEX (Sigma) and 0.5 g/L tetracycline (Solarbio) consecutively for 2 weeks before *Pneumocystis* inoculation. Mice were continuously fed DEX before being sacrificed (6, 7). To explore the function of LPC, the DEX-treated mice were intranasally administered LPC (MedChemExpress) 5 μg each time on day 8, day 10 and day 12 post *Pneumocystis* infection. The experimental protocol was approved by the Capital Medical University Animal Care and Use Committee.

### 4.3 *Pneumocystis* infection

*P. murina* were obtained from American Type Culture Collection (ATCC, Manassas, VA) and were maintained in CB-17 SCID mice as previously reported (6, 7). To construct *Pneumocystis*-infected mouse models, each mouse was intratracheally instilled 1×10^6^ cysts diluted in 100uL sterile PBS, while *Pneumocystis*-uninfected control mice were administered 100 μL sterile PBS (8, 64). Mice were humanely killed under anesthesia at indicated time points. The *Pneumocystis* burden in the middle lobe of right lung was quantified based on the TaqMan qPCR as previously detailedly elaborated (5, 65). In addition, the upper lobe of right lung was fixed in 4% paraformaldehyde and performed H&E staining for histological analysis.

### 4.4 Preparation of single-cell suspensions

Mice were sacrificed under anesthetization at specific time points. Lung tissues were collected, minced and then digested using the Lung Dissociation Kit (Miltenyi Biotech) in combination with the gentleMACS Dissociators (Miltenyi Biotech) in accordance with the instruction manual. After incubation at 37°C for 30 min with shaking, cell suspensions were filtered with 70 μm cell strainers and centrifuged at 300 g for 5 min. Then the resulting pellet was lysed with RBC lysing buffer to remove erythrocytes followed by centrifugation as well as cell counts and viability determination.

### 4.5 scRNA-seq and data analysis

Single-cell libraries were constructed using the 10×Genomics Single Cell 3′ Library & Gel Bead Kit v3 (10×Genomics) and 10×Genomics Chromium Controller (10×Genomics). After quality assessment using Agilent 4200, the libraries were sequenced *via* the Illumina NovaSeq 6000 System (Illumina, San Diego, CA) (performed by CapitalBio Technology Inc., Beijing, China and Novogene Co. Ltd, Beijing, China). To generate raw gene expression matrices, the Cell Ranger (v.4.0.0) was applied. Mouse GRCm38/mm10 and human GRCh38-1.2.0 were used as reference genome respectively.

Then the Seurat (66) package (v.4.1.0) in R software (v.4.1.2) was used for further analysis including quality control and cell clustering. Doublets identified by Scrublet were discarded. For mouse scRNA-seq data, cells detected fewer than 400 genes, greater than 6000 genes or 25000 unique molecular identifiers (UMIs), as well as cells containing more than 10% of reads derived from mitochondrial genome were removed from further analysis. For human BALF cells scRNA-seq data, cells detected fewer than 200 genes or containing more than 20% of reads derived from mitochondrial genome were removed from further analysis. Following quality control, the NormalizeData function was used for data normalization and the top 3000 variable genes were identified *via* the FindVariableFeatures function. To reduce the dimensions, the scaled data was obtained from the ScaleData function and used to perform principal component analysis and t-distributed stochastic neighbor embedding (t-SNE) projections. Lastly, cells were clustered and annotated based on the expressions of known canonical markers.

### 4.6 Differentially expressed genes and pathway enrichment

Differentially expressed genes were identified using the FindMarkers function by Wilcoxon Rank Sum test. Then we selected genes expressed in more than 10% of cells and with a log fold change threshold of 0.1. Genes obtained from comparison between specific cell types were ranked based on their expression. Then GSEA was performed *via* clusterProfiler R package (v.4.2.2) against pathways in GO and KEGG database as well as hallmark gene sets.

To compare the group 2 macrophages in human data and Mmp12^+^ macrophages in mouse data, we generated group 2 macrophages signatures (i.e., upregulated genes in group 2 *versus* group 1&3 macrophages and log_2_ FC > 0.5) and Mmp12^+^ macrophages signatures (i.e., upregulated genes in Mmp12^+^ macrophages *versus* other macrophages and log_2_ FC > 0.5) based on scRNA-seq data respectively. Then we performed transcriptomic comparison *via* GSEA.

### 4.7 Single-cell trajectory analysis

The Monocle2 (67) R package (v.2.22.0) was used to conduct pseudotime analysis and infer the cell lineage developmental trajectories of macrophages and monocytes. Following ordering cells and dimensionality reduction with default parameters, cells were plotted and colored by pseudotime or cell types to visualize using the plot_cell_trajectory function.

### 4.8 Determination of cytokines levels in BALFs

Mice were killed under anesthesia and then lungs were lavaged with sterile PBS. Followed by centrifugation, the supernatants were collected and measured using ProcartaPlex Multiplex Immunoassay (ThermoFisher) based on Luminex platform to detect cytokines and chemokines levels according to manufacturer’s recommendations.

### 4.9 Quasi-targeted metabolomics

BALFs collected from 4 group of mice were centrifuged and 100 μL of supernatants were tested *via* LC-MS/MS system analyses (performed by Novogene Co. Ltd, Beijing, China). After metabolites quantification and annotation using the KEGG, Human Metabolome Database and Lipid maps database, principal component analysis and partial least squares discriminant analysis were performed at metaX software (68).

### 4.10 Immunohistochemistry

We first deparaffinized and rehydrated the paraffin-embedded lung tissue sections and blocked endogenous peroxidase activity. Then we performed antigen retrieval with tris-EDTA buffer pH 9.0. Sections were blocked with goat serum, incubated with either anti-MMP-12 (Proteintech) or anti-CD68 (Proteintech) antibodies, then incubated with secondary antibody Goat Anti-Rabbit IgG H&L (HRP) (Abcam), stained with DAB and counterstained briefly with hematoxylin before microscopic observation.

### 4.11 qPCR verification

Total RNA was extracted from mice lung and then quantified. cDNA was synthesized using PrimeScript RT reagent Kit with gDNA Eraser (TaKaRa) and qPCR was conducted using LightCycler 480 SYBR Green I Master (Roche). Then we calculated gene expression *via* the 2^-ΔΔ^CT method. β-actin was used as a housing-keeping gene. Primers were as follows: *Mmp12* 5’-CCTGCTTACCCCAAGCTGAT-3’ and 5’-ATGTTTTGGTGACACGACGG-3’; *Itgax* 5’-CTGGATAGCCTTTCTTCTGCTG-3’ and 5’-GCACACTGTGTCCGAACTCA-3’; *Irf4* 5’-TCCGACAGTGGTTGATCGAC-3’ and 5’-CCTCACGATTGTAGTCCTGCTT-3’; *Lpcat3* 5’-GACGGGGACATGGGAGAGA-3’ and 5’-GTAAAACAGAGCCAACGGGTAG-3’; *Nr1h3* 5’-CTCAATGCCTGATGTTTCTCCT-3’ and 5’-TCCAACCCTATCCCTAAAGCAA-3’; *Il21* 5’- GGACCCTTGTCTGTCTGGTAG-3’ and 5’- TGTGGAGCTGATAGAAGTTCAGG-3’; *Lif* 5’- ATTGTGCCCTTACTGCTGCTG-3’ and 5’- GCCAGTTGATTCTTGATCTGGT-3’; *Il1b* 5’-GCAACTGTTCCTGAACTCAACT-3’ and 5’-ATCTTTTGGGGTCCGTCAACT-3’; *Il6* 5’-CCCCAATTTCCAATGCTCTCC-3’ and 5’-CGCACTAGGTTTGCCGAGTA-3’; *Tnf* 5’- CGGGCAGGTCTACTTTGGAG -3’ and 5’-ACCCTGAGCCATAATCCCCT-3’.

### 4.12 Macrophages-mediated *Pneumocystis* killing assay

AM-like cells were generated from adult mouse bone marrow as described (27). Briefly, the mouse bone marrow-derived cells were isolated and cultured in the presence of 20 ng/mL GM-CSF and 2 ng/mL TGF-β for 7 days. Then the cell culture medium was replaced with 20 ng/mL GM-CSF, 2 ng/mL TGF-β and 0.1 μM rosiglitazone. At day 9, the adherent macrophages were collected as AM-like cells.

The fungal killing capacity of macrophages was assessed as described in a previous study (69). Briefly, AM-like cells were seeded into a 24-well plate at a density of 2×10^5^ cells per well, and treated with 100 nM DEX for 6 hr. Then these cells were challenged with 8×10^5^ *P. murina* (1:4 ratio) per well. The mRNA expression of *Lif*, *Il1b*, *Il6* and *Tnf*, as well as the *P. murina* burden were assessed by qPCR at 2 hr and 24 hr post infection, respectively.

## Data availability statement

The names of the repository/repositories and accession number(s) can be found below: https://www.ncbi.nlm.nih.gov/geo/, GSE225246; https://ngdc.cncb.ac.cn/gsa-human/, HRA004082.

## Ethics statement

The studies involving human participants were reviewed and approved by the Institutional Review Boards of Beijing Chao-Yang Hospital, Capital Medical University. The patients/participants provided their written informed consent to participate in this study. The animal study was reviewed and approved by the Animal Experiments and Experimental Animal Welfare Committee of Capital Medical University.

## Author contributions

ZT and NS conceived and designed the study. YW, KL, WZ and HY performed the experiments. YL and TL contributed to the collection of clinical specimens. YW and KL analyzed the data, performed bioinformatic analysis and drafted the manuscript. WZ, ZT and NS revised the manuscript. All authors contributed to the article and approved the submitted version.
