# Supplementary material for: Integrated multi-omics analyses reveal the altered transcriptomic characteristics of pulmonary macrophages in immunocompromised hosts with Pneumocystis pneumonia
Source: Front Immunol. 2023 Jun 9;14:1179094. doi: 10.3389/fimmu.2023.1179094 (PMC10289015; doi:10.3389/fimmu.2023.1179094)
Supplement: Supplementary file 1 [file DataSheet_1.docx]

***Supplementary Material***

**I****ntegrated multi-omics analyses reveal the altered transcriptomic characteristics of pulmonary macrophages in immunocompromised hosts with *Pneumocystis pneumonia***

Yawen Wang^1,^^†^, Kang Li^1,†^, Weichao Zhao^1,3^, Yalan Liu^1^, Ting Li^1^, Huqin Yang^1^, Zhaohui Tong^1,*^, Nan Song^2,*^

^1^ Department of Respiratory and Critical Care Medicine, Beijing Institute of Respiratory Medicine and Beijing Chao-Yang Hospital, Capital Medical University, Beijing, 10020, China

^2^ Medical Research Center, Beijing Institute of Respiratory Medicine and Beijing Chao-Yang Hospital, Capital Medical University, Beijing, 10020, China

^3^ Department of Respiratory Medicine, PLA Strategic Support Force Medical Center, Beijing, 100101, China

^†^ These authors contributed equally to this work.

**^*^ Correspondence:**

Zhaohui Tong: tongzhaohuicy@sina.com (ZT); Nan Song: nsong@ccmu.edu.cn (NS)


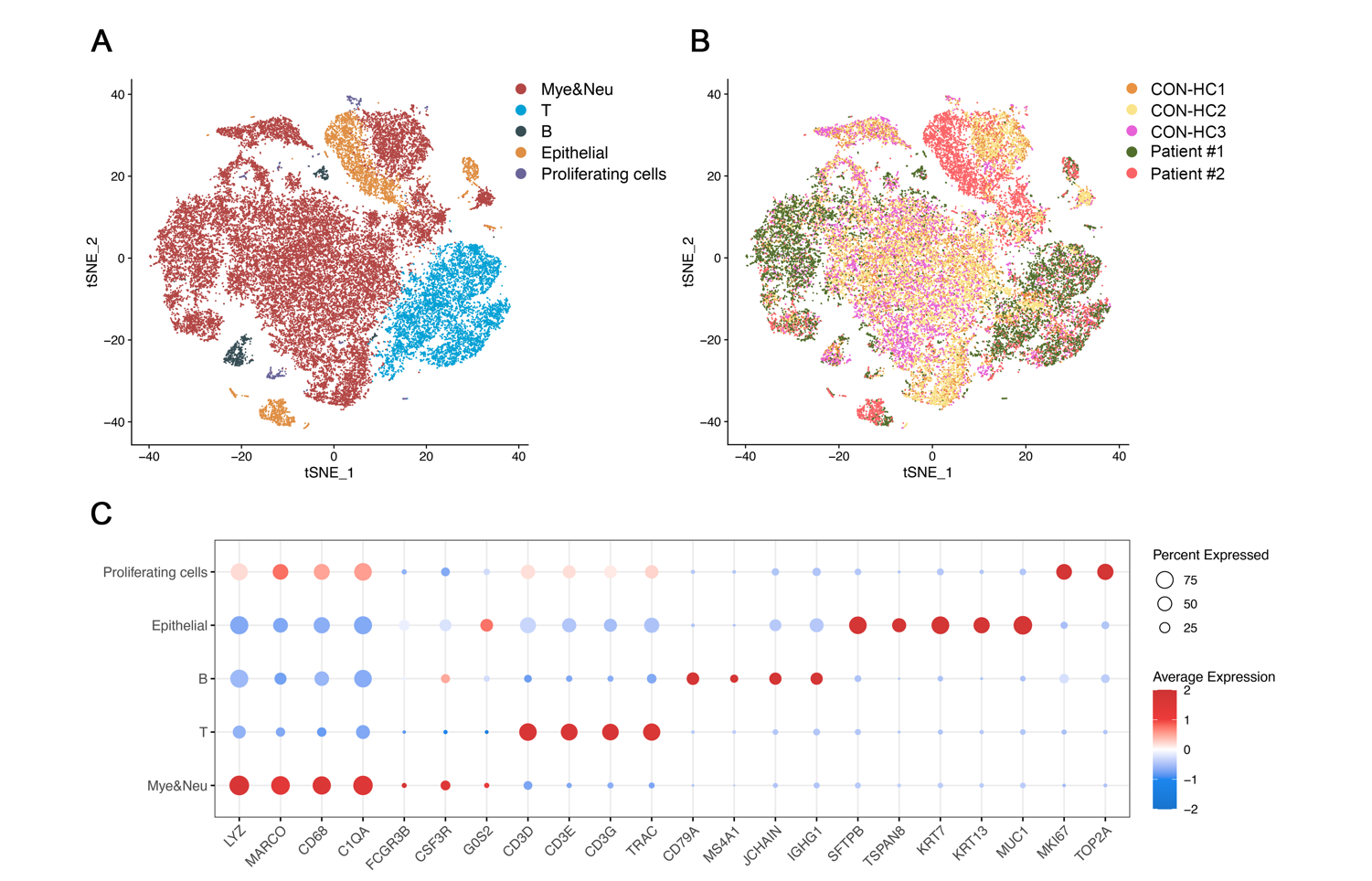


**Supplementary Fig. 1. Cell types recovered in human BALFs from healthy donors and *Pneumocystis*-infected patients using scRNA-seq.**

**A.** t-SNE plot for 38203 cells, color-coded by cell types. **B.** t-SNE plot for 38203 cells, color-coded by donors with 10508 cells from CON-HC1, 9732 cells from CON-HC2, 4959 cells from CON-HC3, 8268 cells from Patient #1 (Non-glucocorticoids) and 4736 cells from Patient #2 (Glucocorticoids). **C.** Dot plot showing the average expression of canonical markers of each cell type. Data were colored based on the gene expression levels.
